# Real-world evidence of patients with metastatic castration-resistant prostate cancer treated with cabazitaxel: comparison with the randomized clinical study CARD

**DOI:** 10.1038/s41391-021-00487-1

**Published:** 2022-01-17

**Authors:** Ronald de Wit, Stephen J. Freedland, Stephane Oudard, Georgi Marinov, Philippe Capart, Austin J. Combest, Ryan Peterson, Ayse Ozatilgan, Alicia K. Morgans

**Affiliations:** 1 Erasmus Medical Center, Rotterdam, The Netherlands; 2 Division of Urology, Cedars-Sinai Medical Center, Los Angeles, CA USA; 3 Section of Urology, Durham VA Medical Center, Durham, NC USA; 4 George Pompidou European Hospital, University of Paris, Paris, France; 5 PPD, Sofia, Bulgaria; 6 Medimix, Miami, FL USA; 7 University of North Carolina at Chapel Hill, Chapel Hill, NC USA; 8 PPD, Wilmington, NC USA; 9 Sanofi, Global Medical Oncology, Cambridge, MA USA; 10 Massachusetts College of Pharmacy and Health Services, Boston, MA USA; 11 Northwestern University Feinberg School of Medicine, Chicago, IL USA

**Keywords:** Cancer therapy, Cancer therapy

## Abstract

**Background:**

The CARD study demonstrated superiority of cabazitaxel over abiraterone/enzalutamide in patients with metastatic castration-resistant prostate cancer (mCRPC) who received prior docetaxel and progressed ≤12 months on the alternative androgen-receptor-targeted agent (ARTA). The objective was to compare characteristics and treatment patterns of patients from a real-world dataset with the CARD population.

**Methods:**

Real-world data were collected from Medimix Live Tracker^TM^, a retrospective, global oncology database of healthcare professional-reported electronic patient medical forms (2001–2019), with data from patients from Europe, USA, Brazil and Japan. The database contained patient, tumor and treatment information for 12,140 patients who received ≥1 line of treatment for mCRPC. A CARD-like cohort included patients treated with docetaxel, prior abiraterone/enzalutamide and cabazitaxel.

**Results:**

A large proportion of patients received ≥2 lines of ARTA (35.1%) with 42% of patients who received a first-line ARTA receiving another ARTA in second line. Of the total patients, 452 were eligible for the CARD-like cohort. Median age of the CARD-like cohort was comparable to CARD (73 vs 70 years). The CARD-like cohort had unfavorable disease characteristics vs CARD: ECOG PS ≥ 2 (45% vs 4.7%); metastasis at diagnosis (46% vs 38%) and Gleason 8–10 (65% vs 57%). More patients in the CARD-like cohort received ARTA before docetaxel (48% vs 39%) and received the first ARTA for >12 months (30% vs 17%) compared with CARD. Despite more patients in the CARD-like cohort receiving the lower 20 mg/m^2^ dose of cabazitaxel (55% vs 21%), cabazitaxel treatment duration was similar (21.9 vs 22.0 weeks).

**Conclusions:**

Sequential use of ARTA was frequent. Results indicate the CARD population is reflective of routine clinical practice and duration of response to cabazitaxel was similar in a real-world population.

## Introduction

Prostate cancer is the second most frequent cancer among men and the fifth leading cause of cancer-related death worldwide [1]. Management of advanced prostate cancer has dramatically evolved over the past 15 years. Treatments approved for metastatic castration-resistant prostate cancer (mCRPC) include taxanes (docetaxel, cabazitaxel), androgen-receptor-targeted agents (ARTAs; abiraterone, enzalutamide), radioisotopes (radium-223), poly-ADP ribose polymerase inhibitors (olaparib and rucaparib), and immunotherapy (sipuleucel-t and pembrolizumab for microsatellite instability high/deficient mismatch repair tumors) [2, 3]. Docetaxel was the first chemotherapy to demonstrate a significant survival benefit for patients with mCRPC based on the TAX-327 Phase III trial [4]. Cabazitaxel was approved for the treatment of patients with mCRPC previously treated with docetaxel, demonstrating overall survival benefits over mitoxantrone in the Phase III TROPIC trial [5]. Studies also demonstrated that cabazitaxel retains its activity in patients whose disease progressed with androgen-signaling-targeted inhibitors [6–8].

The optimal sequencing of available treatments for patients with mCRPC is unknown. Chemotherapy has routinely been reserved for later lines of treatment, and is sometimes not used at all, due to patient health status and patients’ preferences to avoid the side effect profile [9–11]. Thus, in clinical practice, many patients with prostate cancer often receive sequential ARTAs despite evidence that many patients may not benefit from a second alternative inhibitor [12]. Retrospective studies evaluating the sequential use of abiraterone and enzalutamide in patients with mCRPC after treatment with docetaxel have suggested cross-resistance between ARTAs, and two recent prospective studies demonstrate that patients who have progressed on an ARTA are unlikely to respond to a second alternative inhibitor [12–16]. Since multiple treatment options are available for patients with mCRPC, and the optimal sequence of these treatments remains unknown, it is critical to better understand patients’ and physicians’ preferences and how treatment choices may be influenced by patient characteristics, and treatment access [9, 17].

To address the question of optimal sequencing of treatments for mCRPC, the prospective, randomized CARD study (NCT02485691) compared the efficacy and safety of cabazitaxel versus abiraterone or enzalutamide in patients with mCRPC who had previously received docetaxel and who progressed within 12 months on the alternative ARTA. Patients receiving cabazitaxel in this setting had significantly longer imaging-based progression-free survival and overall survival compared with patients receiving abiraterone or enzalutamide, as well as improvements in other secondary endpoints, including prostate-specific antigen response, pain and tumor responses, occurrence of symptomatic skeletal events, and quality of life as measured by the EQ-5D-5L utility index [18, 19]. The incidence of grade ≥3 adverse events was comparable between arms [19].

Prospective, randomized trials enroll highly selected patients with good performance status and well-controlled comorbidities, while patients with poor functional status and greater comorbidity burden are often under-represented. Real-world studies offer the possibility of validating the results of randomized clinical trials by including patient populations reflective of those in routine clinical practice and characterizing trends in healthcare service utilization. Here, we report the findings of a real-world study assessing the treatment sequences received by patients with mCRPC in daily clinical practice. We also evaluated the characteristics of patients receiving cabazitaxel who satisfied the main CARD enrollment criteria (i.e. prior treatment with docetaxel and one ARTA, in any order) to determine whether the population of patients in the CARD study is reflective of the patients with mCRPC seen in routine clinical practice.

## Materials and methods

### Study description

Real-world data available in the Medimix (now Evidera) LiveTracker^TM^ database were used in this study. The LiveTracker^TM^ contains data from electronic patient medical forms entered into the database by a global network of medical oncologists and urologists who were the treating physicians of the respective patients. This retrospective, observational, cohort study focused on patient data entered into the database over the period of January 2019 to December 2019. Treatment sequence for all patients in the dataset was evaluated. We also assessed the characteristics of patients receiving cabazitaxel after docetaxel and one ARTA to compare patient characteristics and the treatment duration of cabazitaxel with the CARD clinical study population. In the CARD study (NCT02485691), cabazitaxel treatment duration was defined as Last dose date - first dose date +21 days [15]. In the cohort analysis, treatment duration was not collected, but patients were classed as on treatment if they received it for ≥1 day and calculated as treatment end date—treatment start date. A new line of therapy was defined as a switch to a new treatment or if the physician had entered the same treatment in two or more consecutive, distinct lines.

### Study patients

The database contains patient characteristics, prescribing physician/institution information, and tumor and treatment information for patients who received at least one line of active treatment for mCRPC between 2001 and 2019. For this analysis, patients with mCRPC treated in France, Germany, Italy, Spain, United Kingdom, USA, Japan and Brazil were included. Data were collected following a cross-sectional retrospective methodology, with no patient identifiers collected. Collected data were in total respect of General Data Protection Regulation and Health Insurance Portability and Accountability Act compliance guidelines. The CARD study was approved by the institutional review board at each center and was conducted in compliance with the principles of the Declaration of Helsinki and Good Clinical Practice guidelines. All patients provided written informed consent.

### CARD-like cohort of patients

In a second step, we restricted the population to patients satisfying the main inclusion criteria of the CARD study to define the CARD-like cohort. This cohort comprised patients with mCRPC who had previously been treated with three or more cycles of docetaxel, had previously received abiraterone or enzalutamide treatment for any duration of exposure (≥1 day), before or after docetaxel therapy, and received cabazitaxel after all previous conditions were met. The use of docetaxel or abiraterone in the context of metastatic hormone-sensitive disease and docetaxel rechallenge were allowed. Patients were excluded from the CARD-like cohort if they had received more than one ARTA prior to cabazitaxel or if they had received prior chemotherapy other than docetaxel for prostate cancer except estramustine. Data analysis was performed on patients with documented treatment durations.

The following data were collected: patient and disease characteristics, history of prostate cancer, baseline laboratory outcomes, details of previous therapies and treatment exposure.

## Results

### Overall treatment trends for mCRPC

**Fig. 1:**
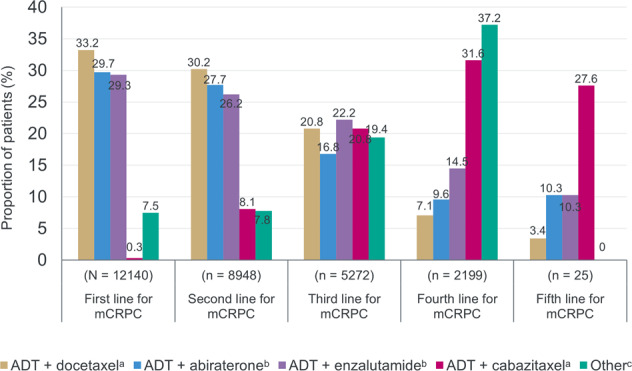
Treatment regimen prescribed by line of therapy. ^a^Treatment may include addition of denosumab. ^b^Treatment may include addition of denosumab or zoledronic acid. ^c^Treatment may include addition of denosumab, active surveillance, ADT, ADT + bicalutamide, ADT + pembrolizumab, ADT + mitoxantrone + prednisone, ADT + sipuleucel-T, ADT + steroids, ADT + radium-223, LHRH + bicalutamide, LHRH agonist + flutamide, pain management, palliative care, abiraterone + radium-223, clinical trial or was unspecified. ADT androgen deprivation therapy, LHRH luteinizing hormone-releasing hormone, mCRPC metastatic castration-resistant prostate cancer.

**Fig. 2:**
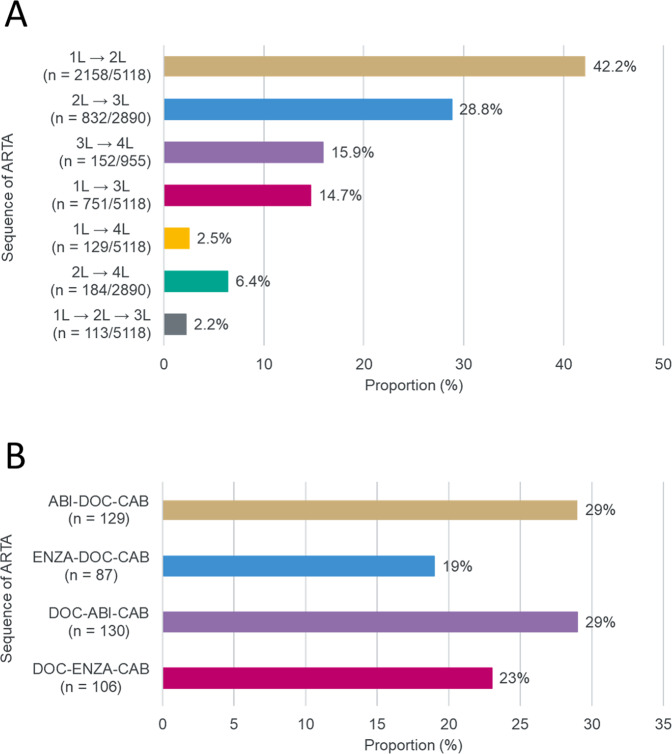
Treatment sequencing in the CARD-like cohort. **A** Treatment sequence of patients in the database who received at least two ARTAs by line of therapy^a^ and **B** most frequent treatment sequences in the CARD-like cohort. ^a^Total number of patients provided represents the number of patients included in the database who have data for the given lines of therapy. ARTA androgen-receptor targeted agent, CAB cabazitaxel, DOC docetaxel, ENZA enzalutamide, L line.

A total of 12,140 patients received at least one line of active treatment for mCRPC between 2001 and 2019; Table 1. The number of patients with available data for each line decreased with advancing lines of treatment (Fig. 1). The proportion of patients receiving ARTAs decreased after the second line of treatment for mCRPC, whereas the proportion of patients receiving cabazitaxel increased (Fig. 1). A large proportion of patients received at least two lines of ARTA treatment (3142 [35.1%]; Fig. 2A). Of the patients who received an ARTA in first line (*n* = 5118), 42% received the same or an alternative inhibitor in the second line. There were 75 patients who received abiraterone in both first and second line. The median break between lines of abiraterone was 30 days (range 0–577 days). There were 125 patients who received enzalutamide in both first and second line. The median break between lines of enzalutamide was 30 days (range 0–394 days). Of the patients who received an ARTA in the second line (*n* = 2890), 29% received the same or an alternative inhibitor in the third line and 16% in fourth line.

**Table 1:** Baseline characteristics.

Characteristic	RWE	CARD-like cohort	CARD study
	All patients (*N* = 12,140)	Cabazitaxel (*n* = 452)	Cabazitaxel (*n* = 129)
Age			
Median, years (range)	72 (34–98)	73 (38–86)	70.0 (46–85)
≥75 years, *n* (%)	4652 (38.5)	180 (39.8)	45 (34.9)
Country, *n* (%)			
Brazil	829 (6.8)	16 (3.5)	-
France	1702 (14.0)	94 (20.8)	23 (17.8)
Germany	1820 (15.0)	67 (14.8)	17 (13.2)
Italy	1180 (9.7)	64 (14.2)	18 (14.0)
Japan	1266 (10.4)	39 (8.6)	-
Spain	1181 (9.7)	66 (14.6)	17 (13.2)
United Kingdom	1400 (11.5)	61 (13.5)	4 (3.1)
United States	2762 (22.8)	45 (10.0)	-
Austria	-	-	7 (5.4)
Belgium	-	-	8 (6.2)
Czech Republic	-	-	10 (7.8)
Greece	-	-	6 (4.7)
Iceland	-	-	4 (3.1)
Ireland	-	-	1 (0.8)
The Netherlands	-	-	10 (7.8)
Norway	-	-	4 (3.1)
ECOG performance status score, *n* (%)			
0–1	8921 (73.5)	247 (54.6)	123 (95.3)
2	2414 (19.9)	151 (33.4)	6 (4.7)
3–4	-	45 (11.9)	-
Liver or lung metastases, *n* (%)	1085 (8.9)	56 (12.4)	21 (16.3)
Type of progression at trial entry, *n* (%)			
PSA only	-	50 (11.1)	11 (8.5)
Imaging-based, with or without PSA progression	-	46 (10.2)	23 (17.8)
Pain, with or without PSA progression	-	-	86 (66.7)
Lack of response/disease progression	-	237 (52.4)	-
Other	-	54 (11.9)	-
Missing data	-	65 (14.4)	9 (7.0)
Disease history			
M1 disease at diagnosis, *n* (%)	3937 (32.7)	209 (46.2)	49 (38.0)
Gleason score 8–10 at diagnosis, *n* (%)	7011 (57.8)	295 (65.2)	73 (56.6)
Previous ARTA, *n* (%)			
Abiraterone	-	259 (57.3)	56 (43.4)
Enzalutamide	-	193 (42.7)	72 (55.8)
Timing of previous ARTA, *n* (%)			
Before docetaxel	-	216 (47.8)	50 (38.8)
After docetaxel	-	236 (52.2)	79 (61.2)
Timing from initiation of previous ARTA to progression, *n* (%)			
≤12 months	-	297 (65.7)	103 (89.2)
>12 months	-	136 (30.1)	22 (17.1)
Unknown	-	19 (4.2)	-
Treatment for mHSPC, *n* (%)			
ADT + docetaxel	-	49 (10.8)	14
ADT + abiraterone	-	19 (4.2)	0

*ADT* androgen deprivation therapy, *ARTA* androgen-receptor-targeted agent, *ECOG* Eastern Cooperative Oncology Group, *mHSPC* metastatic hormone-sensitive prostate cancer, *PSA* prostate-specific antigen, *RWE* real-world evidence.

One hundred and thirteen patients (0.9%) received ARTAs consecutively in first, second and third lines. At some point over the course of their treatment, 2546 (21%) patients received cabazitaxel, predominantly in third and fourth lines of therapy (Fig. 1). Of the 695 patients who received fourth-line cabazitaxel, 78% had already received both abiraterone and enzalutamide.

### Treatment trends for patients with DNA repair mutant mCRPC

Although the number of patients in the database tested for DNA repair mutations (*BRCA1*, *BRCA2* and *ATM*) is unknown, mutations were reported in 649, of which 177 received cabazitaxel. In these patients, median duration of treatment with cabazitaxel was longer than with docetaxel, both in second-line setting (26.1 weeks vs 17.4 weeks) and third-line setting (19.6 weeks vs 8.7 weeks; Table 2).

**Table 2:** Treatment duration in patients with BRCA1, 2 or ATM-mutated mCRPC.

	Docetaxel		Cabazitaxel	
	Patients, *N*	Median treatment duration, weeks (range)	Patients, *N*	Median treatment duration, weeks (range)
First line	210	25.9 (1.0–108.9)	N/A	N/A
Second line	81	17.4 (1.0–56.6)	60	26.1 (1.0–78.3)
Third line	57	8.7 (1.0–86.9)	20	19.6 (1.0–52.1)

Conclusions about mutational frequencies should not be made from this data because the number of patients tested for DNA repair mutations within the cohort is unknown.

*mCRPC* metastatic castration-resistant prostate cancer.

### Patient characteristics in the CARD-like cohort

Of the 12,140 patients with mCRPC included in the database, 452 patients received cabazitaxel after progressing on docetaxel and one ARTA, in any order, and had documented treatment durations. Patient baseline characteristics are summarized in Table 1. The median age for the CARD-like cohort was similar to the CARD study (73 vs 70 years). More patients in the CARD-like cohort had unfavorable disease characteristics compared with the CARD study patients including Eastern Cooperative Oncology Group (ECOG) performance status (PS) score ≥ 2 (45% vs 4.7%), M1 disease at diagnosis (46% vs 38%) and Gleason 8–10 (65% vs 57%). The proportion of patients with visceral metastases compared with CARD was 12% vs 16%. More patients in the CARD-like cohort received a prior ARTA before docetaxel compared with the CARD study (48% vs 39%). Additionally, 57% of patients received abiraterone as a prior ARTA compared with 43% in the CARD study. In the CARD-like cohort, 297 patients (66%) received prior ARTA treatment for ≤12 months compared with 136 patients (30%) who received ARTA treatment for >12 months, which is higher than in the CARD study (17%).

### Treatment sequence and duration in the CARD-like cohort

More patients in the CARD-like cohort received ≥1 cycle of the lower 20 mg/m^2^ dose of cabazitaxel instead of the 25 mg/m^2^ dose used in the CARD study (55% vs 21%; Table 3). The lower dose may have been used as the starting dose in the CARD-like cohort, while the starting dose in the CARD trials was 25 mg/m^2^ for all patients. Despite the larger proportion of patients receiving a lower dose of cabazitaxel in the CARD-like cohort compared with the CARD study, the duration of cabazitaxel treatment received was comparable (21.9 vs 22.0 weeks). In the CARD-like cohort, the proportion of patients receiving cabazitaxel after first-line ARTA and second-line docetaxel was similar to those receiving first-line docetaxel and second-line ARTA (Fig. 2B). Use of granulocyte-colony stimulating factor and other supportive care regimens were not documented in the database.

**Table 3:** Treatment exposure for patients with mCRPC receiving cabazitaxel.

	CARD-like cohort	CARD study
	Cabazitaxel (*n* = 452)	Cabazitaxel (*n* = 129)
Duration of cabazitaxel treatment		
Median duration of cabazitaxel exposure, weeks (range)	21.9 (1.0–117.4)	22.0 (3.0–88.0)
Duration of cabazitaxel treatment in patients who received their first ARTA ≤ 12 months, weeks (range)^a^	21.6 (1.0–117.4)	23.9 (3.0–87.9)
Duration of cabazitaxel treatment in patients who received their first ARTA > 12 months, weeks (range)^a^	25.9 (1.0–108.6)	21.6 (6.0–51.7)
Median number of cabazitaxel cycles, *n* (range)	6 (1–15)^b^	7.0 (1.0–29.0)
Cabazitaxel dose reduction		
Patients with ≥1 cycle administered at reduced dose, *n* (%)	250 (55.3)^c^	27 (21.4)
Cabazitaxel discontinuation		
Patients who discontinued cabazitaxel, *n* (%)	452 (100)	120 (95.2)
Reasons for cabazitaxel discontinuation:		
Disease progression	293 (64.8)	55 (43.7)
Adverse event	-	25 (19.8)
Investigator decision	-	21 (16.7)
Patient request	39 (8.6)	12 (9.5)
Other reason	39 (8.6)	7 (5.6)
To improve quality of life	24 (5.3)	-
Not reported	57 (12.6)	0

*ARTA* androgen-receptor-targeted agent, *mCRPC* metastatic castration-resistant prostate cancer.

^a^19 patients in the CARD-like cohort received ARTA for HSPC but no recorded data for treatment duration.

^b^Prescribed number of cycles.

^c^The number of patients includes those who received cabazitaxel at the lower dose of 20 mg/m^2^.

## Discussion

This real-world evidence study, based on patient data entered into the database over the period of 2001 to 2019, demonstrated that sequential use of ARTAs before chemotherapy initiation is common practice. Here we present a cohort of patients with mCRPC who received cabazitaxel after an ARTA, the same as patients in the cabazitaxel arm of the CARD study. More patients in the CARD-like cohort had aggressive disease characteristics, worse ECOG PS, and received a lower dose of cabazitaxel compared with the CARD population. Despite this, the duration of cabazitaxel treatment was similar for the CARD-like cohort and that reported in the CARD study. This suggests that, although the CARD population may not be completely reflective of patients in routine clinical practice, the results of the CARD study are still relevant to patients in routine clinical practice who have received an ARTA.

According to previously published retrospective analysis from the community practice and Veterans Affairs settings in the US, many patients with mCRPC receive ARTAs in sequence [12, 20]. This may be driven by patient health status or patient and clinician preferences to avoid the expected adverse events related to chemotherapy [9, 11]. Recent evidence from several prospective randomized studies demonstrates that progression on one ARTA is associated with a poor response to a second ARTA, likely due to similar mechanisms of resistance [13, 14, 19, 21]. The CARD study demonstrated that cabazitaxel significantly improved imaging-based progression-free survival and overall survival, as well as quality of life and other clinical outcomes, compared with abiraterone or enzalutamide in patients with mCRPC who had previously received docetaxel and progressed within 12 months on the alternative ARTA. In the CARD study, granulocyte-colony stimulating factor was administered at every cabazitaxel cycle and the incidence of grade ≥3 adverse events was comparable between patients treated with cabazitaxel and ARTAs.

Although clinical trials provide valuable evidence for novel treatments, RWE studies are needed to bridge the data reported in clinical trials and how they translate to routine clinical practice. In randomized clinical trials, patients are selected based on strict eligibility criteria. As a result, patients in routine clinical practice may be older, have more advanced or aggressive disease or comorbidities, any of which could subsequently affect the efficacy and tolerability of treatment [22]. In this study, the CARD-like cohort did have more aggressive disease features. However, the duration of treatment with cabazitaxel was comparable to the CARD study. This suggests that the CARD study, although more selective, is representative of patients in routine clinical practice [19].

RWE studies also help identify treatment patterns [22]. In this study, analysis of treatment patterns suggests that in the recent past, sequential use of ARTAs was common in daily practice. This supports the design of the CARD study and that the practice-changing results of the CARD trial are applicable to real-world clinical practice and support the recent changes in clinical practice guidelines [2, 3, 23, 24].

The database was used to perform a small exploratory analysis of treatment patterns among patients with DNA damage repair mutations. Median treatment duration of cabazitaxel was longer than docetaxel in patients with DNA repair abnormalities in the second- and third-line setting, which may be a result of greater activity in this subgroup of patients known to have a poor prognosis, though our study could not specifically test this possibility. The data surrounding the use of taxanes in patients with DNA-repair mutations are conflicted. Some studies have reported no impact, whereas other studies suggest that these patients do have worse outcomes [22, 25, 26]. Although olaparib has recently been approved in the US for this population, there is growing evidence that these patients may benefit from the use of taxanes at some point during their course of treatment [2, 25]. In two large retrospective, international, observational studies, patients with mCRPC tested for germline DNA damage repair mutations had similar progression-free survival and response rate with docetaxel regardless of whether they did or did not have germline DNA damage repair mutations [26, 27]. In this small, exploratory, real-world evidence analysis, the improved treatment duration of cabazitaxel compared with docetaxel in the second- or third-line treatment of patients with rare mutation subtypes may suggest greater activity of cabazitaxel in these patients with aggressive disease, though the data should still be considered hypothesis generating due to limited patient numbers and our inability to track progression risk. The potentially greater activity of cabazitaxel over docetaxel may be due to greater intratumoral drug concentrations of cabazitaxel, especially in patients who were intrinsically resistant to docetaxel [28, 29]. However, these findings are hypothesis generating, and prospective randomized trials are needed to confirm these data.

The CARD study enrolled patients who had disease progression within 12 months of starting an ARTA [19]. An important consideration is whether or not this is reflective of daily clinical practice. The real-world dataset found that a majority of patients treated with cabazitaxel in similar conditions as CARD received less than 12 months of ARTA therapy. These results suggest that these patients experienced disease progression within 12 months of receiving the first ARTA, though specific progression data were not recorded in the database. This finding is also in agreement with findings of prospective randomized studies. Phase III randomized studies conducted with abiraterone and enzalutamide in chemotherapy-naive patients with mCRPC demonstrated a median time until prostate-specific antigen progression of less than 12 months [30, 31]. In a randomized cross-over study of abiraterone followed by enzalutamide (or the inverse sequence) in patients with newly diagnosed mCRPC, time to progression with the first ARTA was less than 8 months [13]. Lastly, in the PLATO study, median duration of first-line enzalutamide in chemo-naïve mCRPC patients was 9.1 months [14]. However, not surprisingly, the number of patients receiving prior ARTA treatment for a duration >12 months was higher in the real-world dataset compared with the CARD study due to the eligibility criteria of CARD requiring that participants have disease progression within 12 months of treatment with one ARTA. Progression was defined by biochemical and/or radiologic and/or clinical progression. In daily clinical practice, patients may continue to receive ARTAs despite prostate-specific antigen progression until unambiguous progression, i.e. radiologic and/or pain progression. Since many physicians do not regularly image their patients at this stage of the disease, ARTAs may be discontinued when severe pain develops rather than at first radiographic progression, a point at which patients frequently experience deterioration of performance status. This may contribute to the higher rate of poor performance status (ECOG PS ≥ 2: 45%) in the CARD-like cohort compared with the CARD study (ECOG PS ≥ 2: 4.7%).

A small proportion of patients received the same ARTA for both first- and second-line treatment. This is not recommended by ESMO or NCCN, either presently or before the practice changing results of the CARD study [15, 21, 23]. Given the short median time between first and second line in this population (30 days), and that some patients had an interval of 0 days, it may be that the change in line was recorded in error. Alternatively, it may be that a new line was recorded as treatment was continued despite indicators of disease progression. There are many factors that could be involved in this observation and due to the limitations of the database, further studies would be needed to investigate further. There are limitations to the study that should be considered when interpreting the results. First, the data available were provided by the clinician and no data audit was performed. This is a common limitation of real-world studies. Additionally, confirmation of progressive disease on the therapy preceding cabazitaxel was not always possible due to the input method used in the database. Although the time to treatment switch was recorded in the database, this was not equivalent to time to progression as was recorded in the CARD study. ECOG PS was recorded at patient data entry only (not necessarily at initiation of cabazitaxel), which is also different to the CARD study that recorded ECOG PS at randomization. As such, the proportion of patients with poor ECOG PS at the time of treatment with cabazitaxel may be greater than what is reported, making an even greater disparity between the CARD-like cohort and the actual CARD study. In light of this, the similar treatment duration between the CARD-like cohort and the actual CARD study is even more noteworthy. Poor ECOG PS due to cancer or other comorbidities could not be differentiated in the database, while only patients with ECOG PS 2 due to prostate cancer could be enrolled in the CARD study. The population of the CARD-like cohort is also not a direct copy of the CARD population as the treatment landscape has changed since CARD enrolment to include PARP inhibitors (olaparib, and rucaparib) for patients with DNA repair abnormalities. Randomized clinical trials are needed to further clarify the place of cabazitaxel in such patients. Differences exist between the clinic and clinical trials in patients’ assessment and treatment. For example, in the real-world cohort, imaging was collected less frequently compared with the CARD study and no data on imaging timing were available, the use of granulocyte-colony stimulating factor was not recorded, and no details were provided on pain management. Finally, this study focused on patient baseline characteristics and treatment durations, but efficacy data were not reported.

In summary, these results indicate that the CARD study population is reflective of patients receiving care in the real-world setting. Additionally, cabazitaxel treatment duration in the CARD study is similar to that observed in daily practice, despite patients in the real world having more aggressive disease characteristics and poorer performance status at baseline. These data highlight the frequent use of sequential ARTAs in this real-world population, despite evidence of shared mechanisms of resistance. As recommended by international guidelines, treatment with cabazitaxel should be the preferred choice in patients with mCRPC with progression of disease after treatment with docetaxel and an ARTA in lieu of treatment with a subsequent ARTA.
